# Antenatal Depressive Symptoms and Computerized Cardiotocography Parameters: An Observational Prospective Study

**DOI:** 10.3390/jcm15218705

**Published:** 2026-11-07

**Authors:** Marco La Verde, Mario Luciano, Mario Ardovino, Francesca Caparrotti, Franca Cerqua, Mario Fordellone, Bianca Della Rocca, Carlotta Brandi, Davide Pisani, Marco Torella

**Affiliations:** 1 Department of Woman, Child and General and Specialized Surgery, University of Campania “Luigi Vanvitelli”, 80138 Naples, Italy; francesca.caparrotti@studenti.unicampania.it (F.C.); franca.cerqua@unicampania.it (F.C.); davidepisani94@gmail.com (D.P.); marco.torella@unicampania.it (M.T.); 2 Department of Psychiatry, University of Campania “Luigi Vanvitelli”, 80138 Naples, Italy; mario.luciano@unicampania.it (M.L.); dellarocca.bianca@gmail.com (B.D.R.); carlotta.brandi@hotmail.com (C.B.); 3 Department of Obstetrics and Gynecology, Ospedale S.G. Moscati, 83100 Avellino, Italy; mario.ardovino@aornmoscati.it; 4 Medical Statistics Unit, University of Campania Luigi Vanvitelli, 80138 Naples, Italy; mario.fordellone@unicampania.it

**Keywords:** depression, cardiotocography, heart rate, fetal, pregnancy trimester, third, psychiatric status rating scales

## Abstract

**Introduction**: The present study aims to investigate the possible computerized cardiotocography (cCTG) parameter alteration in patients with elevated antenatal depressive symptoms, evaluated in the third trimester of pregnancy through the Edinburgh Postnatal Depression Scale (EPDS). **Methods**: We conducted a prospective observational study from 1 August 2024 to 20 August 2025. All pregnant women (after 30 gestational weeks) were included and underwent cCTG and the Edinburgh Postnatal Depression Scale (EPDS) evaluation. Based on their EPDS scores, pregnant women were divided into low (score < 10) or high depressive symptom (score ≥ 10) groups. For all included pregnant women, demographic characteristics and cCTG parameters were recorded. The baseline characteristics and cCTG parameters were compared between groups. Short-term variation (STV) difference represented the primary outcomes. **Results**: A total of 205 pregnant women were enrolled (median gestational age was 38.1 weeks). Demographic, obstetric, or fetal characteristics were homogenous between the groups. There were no cCTG parameter differences between high- and low-EPDS groups: baseline fetal heart rate (*p* = 0.743), short-term variability (*p* = 0.663), long-term variability (*p* = 0.550), accelerations (*p* = 0.893), and fetal movements (*p* = 0.275). The Hodges–Lehmann difference in STV was 0.20 ms (95% CI −0.60 to 1.00), and with excluded differences larger than ±1 ms. EPDS score and cCTG parameter correlations were weak (|ρ| ≤ 0.14). **Discussion:** Maternal depressive symptoms, in late gestation, were not associated with cCTG parameter alterations. These results excluded differences larger than 1 ms but additional studies are needed to explore the fetal autonomic changes related to the maternal depressive symptoms.

## 1. Introduction

The perinatal period is a critical time for mental health disorders, especially perinatal depression (PND). The Diagnostic and Statistical Manual of Mental Disorders—5th edition (DSM-5) defines PND as a major depressive episode occurring during the peripartum period [1]. PND differs from common maternity blues, which can cause crying and irritability in 70% of mothers and resolve spontaneously within two weeks [2]. Depressive symptoms affect around 36.2% of pregnant women [3]. These estimates are conservative, as the condition is often underestimated or underdiagnosed [4]. The Edinburgh Postnatal Depression Scale (EPDS) was adopted to screen PND [5,6]. EPDS consists of 10 items that evaluate the mental state of the patient. Each response is assigned a score, and the total score indicates if a psychiatric evaluation is necessary [6]. The pathogenesis of PND is multifactorial and not yet fully understood and involves environmental as well as biological factors [7,8]. Previous studies showed a link between PND and lower educational level, poor economic status of families, history of mental illness, domestic violence, perinatal smoking or drinking, and multiparity [9]. Biological factors, such as vitamin D, brain-derived neurotrophic factors (BDNFs) and IL-6, have also been associated with an increased risk of PND [10,11]. In the post-partum period, maternal depression represents a risk factor for the child, including behavioral problems and cognitive development disorders [12]. Even in the prenatal period, maternal stress, anxiety, and depression affect the development of the offspring with a significant risk of psychopathology [13]. Cardiotocography (CTG) represents the standard tool for antenatal and intrapartum fetal surveillance [14]. CTG records the fetal heart rate (FHR) and the uterine contractile activity [14,15]. To reduce the intra- and inter-operator variability in CTG analysis, computerized cardiotocography (cCTG) was introduced [16]. This technology provides an objective analysis of the FHR pattern, including short-term variation (STV), a necessary parameter in the management of fetal growth restriction (FGR) [17]. Maternal stress is associated with cortisol and catecholamine levels [18]. This effect could impact FHR variability, accelerations, and cCTG patterns [19]. In this context, maternal psychological state could impact the cCTG variables and the biological plausibility for this link is supported by data. No prospective studies have specifically focused on the antepartum cCTG pattern in the late third trimester and its possible association with maternal psychosocial status, evaluated with the EPDS scores. Our study aimed to address this gap by comparing cCTG variables between pregnant women with and without elevated depressive symptoms.

## 2. Methods

### 2.1. Study Design and Participants

We conducted a prospective observational study at the University of Campania “Luigi Vanvitelli”, Naples, Italy, from 1 August 2024 to 20 August 2025. A portion of the patients were also enrolled in another study that explored the link between maternal perception of fetal movements and cCTG parameters; these two studies had different outcomes, and their results did not overlap [20]. Maternal psychological status was assessed with the EPDS and cCTG during late pregnancy. We followed the STROBE Statement to report observational studies [21]. Consecutive pregnant women undergoing outpatient cCTG at our hospital were enrolled after giving written and oral informed consent; the outpatient cCTG indication followed the routine third-trimester fetal indication. At our institution (second-level referral center), outpatient cCTG is offered as a routine antenatal test to all pregnant women, at term. Our study design did not introduce specific indications. Eligibility criteria were: (1) single pregnancy; (2) gestational age ≥ 30 weeks; (3) maternal age ≥18 years; and (4) written informed consent. Exclusion criteria were: (1) multiple pregnancies; (2) structural or aneuploidy fetal malformations; (3) previous psychiatric disorders requiring medical treatment that could modify the cCTG parameters [22,23]; and (4) use of drugs with FHR impact such as β-blockers. Women with obstetric comorbidities (gestational diabetes, hypertensive disorders, or fetal growth restriction) were not excluded.

### 2.2. Maternal Psychological Assessment

Maternal psychological distress was measured with the EPDS. The EPDS was completed before the cCTG test in a silent, private room. An EPDS cutoff score of 10 or higher was adopted to identify elevated depressive symptoms. This cutoff is consistent with the previous literature, as it serves as a screening test able to identify women with elevated depressive symptoms and a high risk of PND [24,25,26]. After this screening approach, a psychiatric consultation is necessary to determine if a patient meets the DSM-5 criteria for perinatal depression.

### 2.3. Computerized Cardiotocography

Fetal evaluation was carried out with a Sonicaid Team 3 version 8.7 (Huntleigh Healthcare Ltd., Cardiff, UK). Parameters were extracted automatically by the Dawes–Redman algorithm and no patients were excluded for signal loss. The physician did not know the EPDS score. Each cCTG recording lasted at least 20 min (at the clinician’s discretion) with pregnant women in a semi-reclining position, during the morning outpatient clinic, after breakfast. All the cCTG parameters were recorded: FHR baseline, number of accelerations and decelerations, fetal movements per hour (FM/h), high and low episodes, short-term variability (STV), long-term variability (LTV), cCTG duration, and signal loss.

### 2.4. Data Collection and Statistical Analysis

The main objective was to study the fetal heart rate patterns obtained through computerized CTG among the patients with high depressive symptoms. This objective was evaluated primarily through the STV comparison and, secondarily, through the comparison of the remaining cCTG parameters. The analysis hierarchy (with STV as the primary outcome and other parameters as secondary outcomes) was not pre-registered and was formalized during the peer review process, and this decision followed the clinical relevance of STV in the clinical management of the high-risk pregnancy. The distribution of continuous variables was assessed using the Shapiro–Wilk test and visual inspection of histograms and Q–Q plots, and continuous variables were reported as median and interquartile ranges (IQRs). Categorical variables are reported as percentages. Inter-group analyses (EPDS category <10 versus EPDS ≥10) were performed with the Wilcoxon rank-sum test for continuous variables and Fisher’s exact test for categorical variables. Spearman’s rank correlation coefficients with 95% bootstrap confidence intervals were adopted to study the associations between EPDS score and cCTG parameters. *p*-values < 0.05 were considered statistically significant. All the statistical analyses were performed with R Studio Statistical software version 4.1.3 (R Foundation for Statistical Computing, Vienna, Austria). The a priori sample-size estimation was based on a significance test for Pearson’s correlation between the EPDS and continuous cCTG parameters, assuming a two-tailed alpha level of 0.05 and a statistical power of 80%. With a total sample-size of at least 194 participants, the study is powered to detect a minimum effect size of |r|0.20, which corresponds to a small-to-moderate effect according to Cohen’s conventions. This calculation referred to the correlation analysis and was the only prospective power calculation; the study was not prospectively powered for the between-group comparison. A sample-size sensitivity analysis therefore estimated the minimum detectable standardized mean difference for the observed allocation ratio, assuming a two-sided two-sample *t* test, α = 0.05, and 80% power. An exploratory two-one-sided test (TOST) equivalence analysis for STV used Welch unequal-variance standard errors and a margin of ±1 ms. This margin was defined on clinical background: a 1 ms difference is below the clinical STV cutoff (2.6–3.0 ms in the TRUFFLE study [27]). The analysis is exploratory and its results should be interpreted cautiously. As a sensitivity analysis, multivariable linear regression analyses were performed for each cCTG parameter in each group and adjusted for gestational age, maternal age, pre-pregnancy BMI, smoking status, fetal sex and obstetric comorbidities. The study was conducted in accordance with the ethical guidelines of the Declaration of Helsinki and was approved by the Ethics Committee of the University of Campania “Luigi Vanvitelli” (protocol number 98 of 28 February 2019).

## 3. Results

**Figure 1:**
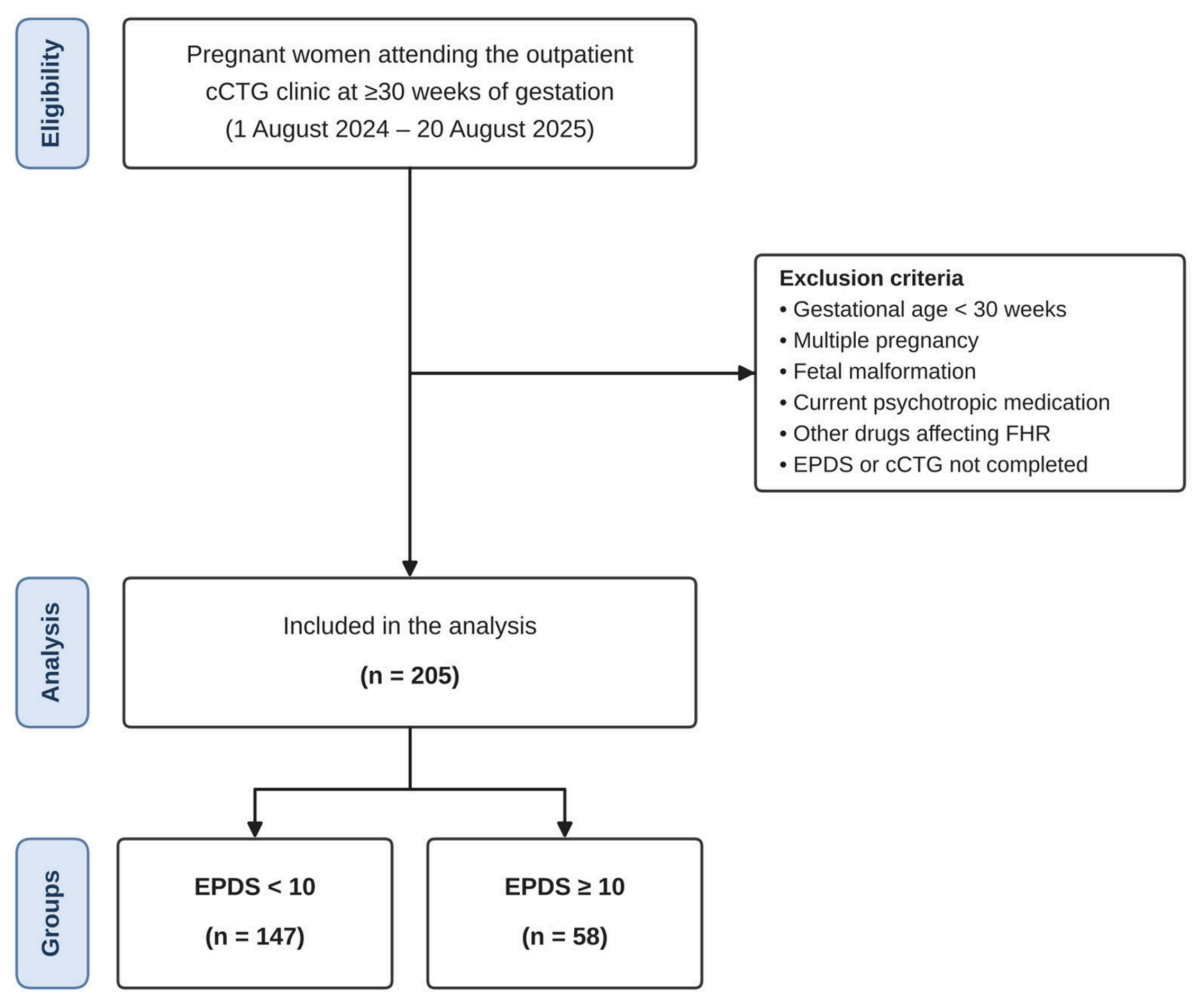
STROBE flow diagram of the study population.

A total of 205 pregnant women were included and categorized in two groups based on EPDS scores: the first group (147) had an EPDS < 10 and the second (58) had an EPDS ≥ 10 (Figure 1). Demographic and clinical features between the two groups were comparable (Table 1). Maternal age (median 31.0 years, IQR 28.00–35.00, versus 30.0 years, IQR 26.25–34.00, *p*-value of 0.39), gestational age in assessment (38.14 weeks, 37.29–39.00, versus 38.50 weeks, 37.29–39.25, *p*-value of 0.55), pre-pregnancy BMI (24.39, 21.56–28.37, versus 24.71, 22.07–29.14, *p*-value of 0.38), or maternal weight (73.0 kg, 68.0–87.0 versus 75.0 kg, 70.0–90.0, *p*-value of 0.18). Distribution of rates of pregnancy by ethnicity, mode of conception, fetal sex, smoking and gestational weight gain did not significantly differ among the EPDS strata (all *p* > 0.05). The majority of pregnancies included were uncomplicated pregnancies, and obstetric comorbidities were present only in 21% of the included pregnancies.

Analyzing the cCTG parameters in the two groups, EPDS < 10 and EPDS ≥ 10 (Table 2), no statistically significant differences were found, and Hodges–Lehmann differences were near to zero. The median CTG duration was 40.0 min (HL difference 0.00 min, 95% CI −3.00 to 2.00; *p* = 0.999), with similar signal loss (0.30% versus 0.30%; *p* = 0.975) and basal FHR (137.00 versus 137.00 bpm; HL −1.00, 95% CI −4.00 to 2.02; *p* = 0.743). FM per hour (30.00 versus 23.30; HL −4.00, 95% CI −10.70 to 3.45; *p* = 0.275), accelerations (10.0 versus 9.0; HL 0.00, 95% CI −1.00 to 1.00; *p* = 0.893), decelerations (*p* = 0.939), and high- and low-variation episodes (*p* = 0.302 and *p* = 0.849) did not show statistically significant differences. LTV (46.05 versus 48.50 ms, HL 1.30, 95% CI −2.90 to 5.40; *p* = 0.550) and STV (8.70 versus 9.30 ms, HL 0.20 ms, 95% CI −0.60 to 1.00; *p* = 0.663) also did not show differences among the groups. The exploratory equivalence analysis showed an STV mean difference of 0.13 ms (90% CI −0.60 to 0.87), which was within the ±1 ms margin (TOST *p* = 0.027). No adjusted association between EPDS group and cCTG parameters was observed in the multivariable models (Supplementary Table S1).

**Figure 2:**
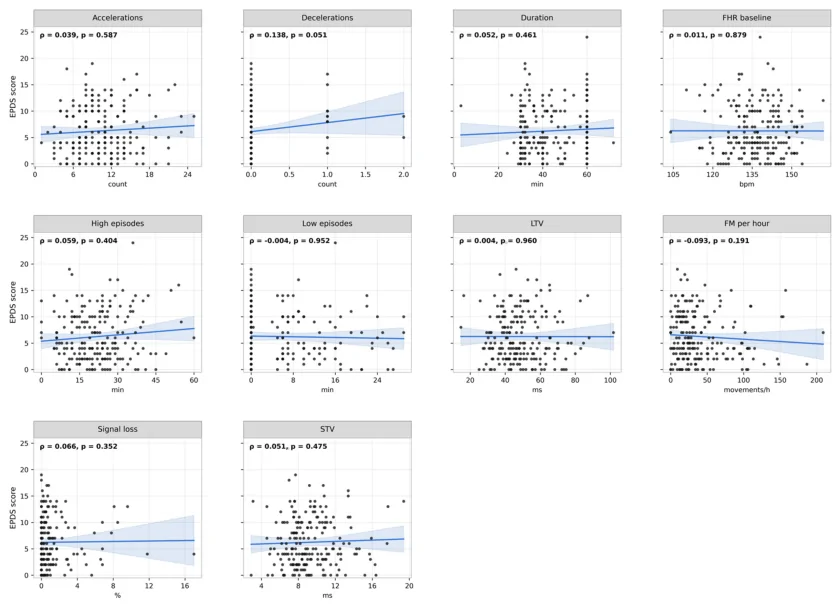
Scatter plot matrix of EPDS score and continuous cCTG parameters.

Figure 2 shows the scatter plot matrix of the EPDS score and the cCTG parameters. All the variables were evaluated, including FHR baseline, number of accelerations and decelerations, FM/h, high and low episodes, STV, LTV, cCTG duration and signal loss. Correlations between EPDS scores and the cCTG parameters were weak (|ρ| ≤ 0.14, Figure 2).

## 4. Discussion

In this prospective study, maternal depressive symptoms, assessed with the EPDS in late gestation, were not associated with significant changes in the cCTG parameters. Several studies have investigated the association between maternal psychological distress and fetal autonomic regulation. Maternal depression and stress may dysregulate the hypothalamic–pituitary–adrenal axis, leading to an increased secretion of cortisol and changes in the sympathetic–parasympathetic balance; these mechanisms could impact fetal heart regulation and fetal neurodevelopment [28]. Experimental and observational studies have associated prenatal depressive symptoms with decreased FHR variability, supporting the idea that maternal affective alteration may reflect intrauterine fetal autonomic regulation [29]. In addition, fetal heart rate variability appeared to be related to fetal neurobehavioral development and postnatal heart self-regulation [30]. The lack of differences in computerized cardiotocography parameters evidenced in our cohort appeared to be discordant with this evidence. However, methodological and conceptual differences are present. The FHR correlation with maternal depressive symptoms was explored through fetal external stimuli [29]. These studies typically investigated beat-to-beat heart rate variability, which reflects rapid autonomic changes and is more sensitive to subtle alterations than average parameters obtained from cCTG [30]. In comparison, cCTG parameters reduced the intra- and inter-observer difference and enabled the clinical evaluation of fetal well-being [31]. In addition, we evaluated the FHR in the third trimester of pregnancy, a period with fetal late autonomic maturation and greater stability of FHR [32]. At this stage of pregnancy, fetal adaptive and compensatory mechanisms improved and could mitigate the maternal psychological distress impact. Severity and clinical depressive symptoms also need consideration. In our cohort, PND risk was evaluated using EPDS screening, without a clinical diagnostic interview. The EPDS quantifies the PND risk and captures a wide range of depressive symptom severity [33]. Maternal abnormalities in autonomic states, especially maternal heart variability, were already explored in women with perinatal depressive symptoms [34]. Different studies investigated maternal heart rate variability associations with post-partum depressive and anxiety symptoms [35]. A scoping review highlights considerable heterogeneity among studies investigating the association between perinatal depression and heart rate variability, varying in the timing, measurement procedures, and population characteristics, which limited the synthesis and interpretation of findings [34]. None of these articles explored the maternal impact on the clinical evaluation of the FHR. cCTG is applied for the objective evaluation of fetal well-being, particularly for fetuses with fetal growth restriction (FGR) [36]. The absence of statistical significance does not necessarily mean that maternal depressive symptoms have no biological effect on the fetal autonomic system. To be precise, our findings excluded differences larger than 1 ms. The STV values shown in our study agree with the STV values observed in the literature [37] and are far from the STV level of increased stillbirth cutoff [27].

The literature shows that PND is linked to different maternal, neonatal, and childhood outcomes, including the reduction in mother–infant interactions, and recent research has suggested that integrated psychological, neuroendocrine, and physiological factors should be considered to fully grasp the influence on maternal–fetal relationships [38,39]. Nevertheless, screening for PND remains necessary to establish links with maternal morbidity, adverse obstetric outcomes, and long-term offspring development [29]. These results reinforce the idea that maternal psychological well-being and fetal surveillance during pregnancy require separate management and that they are complementary but distinct elements of perinatal care. In conclusion, three messages come from this study. First, no clinical association was found between EPDS score and cCTG parameters. Second, this finding did not exclude subtle alterations in fetal autonomic regulation. Third, this finding did not reduce the importance of early detection and treatment of PND.

### 4.1. Strengths

Our study presents several strong aspects. First, it has a prospective design. Second, EPDS represents a validated and internationally accepted screening [30]. Third, FHR was evaluated through cCTG parameters, representing an innovative and objective analysis. Fourth, the exclusion of pregnant women that were receiving psychotropic drugs reduced a possible selection bias related to the cCTG modifications associated with these drugs.

### 4.2. Limitations

This study also presents several limitations that should be considered. First, EPD only represents a screening questionnaire, without confirmation through a structured psychiatric interview or DSM-5 diagnostic assessment. Our study did not present data from the psychiatric interview. As a result, a sub-analysis of the post-partum depression cases was lacking. Second, EPDS scores relate to symptoms present at a single point in the patient’s clinical history and do not capture the entire pregnancy duration or variations during pregnancy [40]. Third, we applied exclusion criteria to reduce major sources of confounding, but other cofounding factors, such as socioeconomic status, acute stress, sleep, maternal anxiety, substance exposure, medication use, or subclinical psychiatric conditions, were not systematically collected and they may still have influenced the results. This unavailable data could represent a possible bias. However, confounding factors could impact the results in either direction, and the main factors that impact the FHR patterns (such as gestational age, smoking, BMI and obstetric comorbidities) were similar among the two groups. Fourth, our study assessed maternal depressive symptoms and cCTG parameters only once, near term (38.1 weeks, IQR 37.3–39.1). Our findings are limited to the late third trimester and term of pregnancy and are not generalizable to all pregnancies. Consequently, our study did not explore if previous maternal psychological changes happen before the single EPDS evaluation. Moreover, fetal autonomic responses changed following the gestational age, and a single assessment at term of pregnancy cannot capture these changes. In addition, cCTG may not be sensitive enough to detect small changes in fetal reactivity, which have been demonstrated in experimental studies using vibroacoustic or vocal stimulation [30]. No cortisol levels or other biochemical markers were evaluated, which are factors potentially involved in these conditions. Future studies that combine psychological assessments with biological stress markers and longitudinal maternal follow-up will be performed to improve our findings.

## 5. Conclusions

In this cohort, elevated antenatal EPDS scores in late pregnancy were not associated with detectable differences in routine computerized CTG parameters. This does not exclude subtle effects on fetal autonomic regulation or effects occurring earlier in pregnancy.

## Figures and Tables

**Table 1:** Baseline clinical characteristics of patients stratified for EPDS class.

	EPDS			
Characteristic	<10, N = 147 ^a^	≥10, N = 58 ^a^	Overall, N = 205 ^a^	*p*-Value ^b^
Ethnicity				0.66
Caucasian	124.0 (84.4%)	51.0 (87.9%)	175.0 (85.4%)	
Other	23.0 (15.6%)	7.0 (12.1%)	30.0 (14.6%)	
Age	31.00 (28.00, 35.00)	30.00 (26.25, 34.00)	31.00 (27.00, 35.00)	0.39
Gestational age	38.14 (37.29, 39.00)	38.50 (37.29, 39.25)	38.14 (37.29, 39.14)	0.55
Onset pregnancy				0.19
IVF	6.0 (4.1%)	0.0 (0.0%)	6.0 (2.9%)	
Spontaneous	141.0 (95.9%)	58.0 (100.0%)	199.0 (97.1%)	
Maternal weight	73.00 (68.00, 87.00)	75.00 (70.00, 90.00)	74.00 (68.00, 88.00)	0.18
Pre-pregnancy BMI	24.39 (21.56, 28.37)	24.71 (22.07, 29.14)	24.44 (21.64, 28.69)	0.38
Weight gain	10.00 (8.00, 13.00)	11.00 (9.00, 14.00)	10.00 (8.00, 13.00)	0.45
Fetal sex				0.56
Female	75.0 (51.0%)	27.0 (46.6%)	102.0 (49.8%)	
Male	68.0 (46.3%)	28.0 (48.3%)	96.0 (46.8%)	
Unknown	4.0 (2.7%)	3.0 (5.2%)	7.0 (3.4%)	
Smoker				0.34
No	132.0 (89.8%)	49.0 (84.5%)	181.0 (88.3%)	
Yes	15.0 (10.2%)	9.0 (15.5%)	24.0 (11.7%)	
Comorbidities ^b^				
GDM	21 (14.2%)	9 (15.5%)	30 (14.6%)	0.82
Pre-GDM	4 (2.7%)	0 (0.0%)	4 (1.9%)	0.20
GH	4 (2.7%)	1 (1.7%)	5 (2.4%)	0.67
Pre-GH	2 (1.3%)	1 (1.7%)	3 (1.4%)	0.67
FGR	2 (1.3%)	2 (3.4%)	4 (1.9%)	0.33

^a^ Median (IQR) or frequency (%); ^b^ total number and (frequency, %); GDM, gestational diabetes; GH, gestational hypertension; FGR, fetal growth restriction; ^b^ *p*-value: Fisher’s exact test; Wilcoxon rank-sum test.

**Table 2:** cCTG parameter by EPDS class with Hodges–Lehmann differences (95% CI).

	EPDS			
Characteristic	EPDS < 10 Median (IQR)	EPDS ≥ 10 Median (IQR)	HL Difference (95% CI)	*p*-Value
Duration (min)	40.00 (32.00, 60.00)	40.00 (33.50, 52.00)	0.00 (−3.00, 2.00)	0.999
Signal loss (%)	0.30 (0.00, 1.00)	0.30 (0.00, 0.80)	0.00 (0.00, 0.10)	0.975
FM per hour	30.00 (16.42, 57.85)	23.30 (13.55, 45.35)	−4.00 (−10.70, 3.45)	0.275
FHR baseline (bpm)	137.00 (130.25, 144.00)	137.00 (130.00, 143.00)	−1.00 (−4.00, 2.02)	0.743
Accelerations	10.00 (7.00, 13.00)	9.00 (8.00, 12.00)	0.00 (−1.00, 1.00)	0.893
Decelerations	0.00 (0.00, 0.00)	0.00 (0.00, 0.00)	0.00 (0.00, 0.00)	0.939
High episodes	20.00 (14.00, 27.75)	24.00 (14.00, 30.00)	2.00 (−1.00, 6.00)	0.302
Low episodes	0.00 (0.00, 10.00)	0.00 (0.00, 9.00)	0.00 (0.00, 0.00)	0.849
LTV	46.05 (39.38, 57.03)	48.50 (39.20, 57.45)	1.30 (−2.90, 5.40)	0.550
STV (ms)	8.70 (7.50, 10.78)	9.30 (7.55, 10.65)	0.20 (−0.60, 1.00)	0.663

HL = Hodges–Lehmann; IQR = interquartile range. Two-sided Mann–Whitney/Wilcoxon rank-sum *p*-values.

## Data Availability

The data presented in this study are available on request from the corresponding author due to privacy and ethical restrictions.

## Supplementary Materials

The following supporting information can be downloaded at https://www.mdpi.com/article/10.3390/jcm15218705/s1: Table S1: Adjusted associations between EPDS group and cCTG parameters.
