# Supplementary material for: Antenatal Depressive Symptoms and Computerized Cardiotocography Parameters: An Observational Prospective Study
Source: J Clin Med. 2026 Nov 7;15(21):8705. doi: 10.3390/jcm15218705 (PMC13764067; doi:10.3390/jcm15218705)
Supplement: Supplementary file 1 [file jcm-15-08705-s001.zip › jcm-4590709-supplementary.pdf]

Table S1. Adjusted associations between EPDS group and cCTG parameters

Characteristic	Adjusted β (95% CI)	p-value
Short-term variability (STV), ms	+0.303 (-0.643, +1.249)	0.530
Long-term variability (LTV)	+1.488 (-3.069, +6.046)	0.522
FHR baseline, bpm	-1.322 (-4.703, +2.060)	0.444
Fetal movements per hour	-5.327 (-17.334, +6.679)	0.384
Accelerations	+0.234 (-1.073, +1.542)	0.725
Decelerations	+0.003 (-0.087, +0.094)	0.943
High episodes	+2.558 (-1.051, +6.168)	0.165
Low episodes	-0.877 (-3.233, +1.478)	0.465
Recording duration, min	-0.624 (-4.474, +3.225)	0.751
Signal loss	+0.048 (-0.664, +0.760)	0.894
